# Comparing the benefits of Cognitive Stimulation Therapy with an adapted collaborative version on traditional outcomes, loneliness, and socioemotional skills: a pilot study

**DOI:** 10.3389/fpsyg.2025.1607552

**Published:** 2025-09-10

**Authors:** Riccardo Domenicucci, Elena Carbone, Enrico Sella, Carmen Belacchi, Michela Sarlo, Erika Borella

**Affiliations:** ^1^Department of General Psychology, University of Padova, Padova, Italy; ^2^Department of Communication Sciences, Humanities and International Studies, University of Urbino Carlo Bo, Urbino, Italy

**Keywords:** cognitive stimulation therapy, collaboratory activities, dementia, loneliness, theory of mind, definitional competence of emotions

## Abstract

**Background:**

Cognitive Stimulation Therapy (CST) is among the psychosocial interventions with strongest evidence of efficacy for the treatment of dementia. It has been argued that people with dementia (PwD) can also benefit from collaboration and teamwork with peers. The aim of this pilot study was to evaluate the benefits of a new Collaborative CST (C-CST) protocol, vis-à-vis the Standard CST (S-CST), on traditional and underexplored, psychosocial and socioemotional outcomes.

**Methods:**

In this single-blind randomized controlled trial, we enrolled a convenience sample of 28 PwD (mean age = 87 ± 5.74 years) from six Italian residential facilities and randomly allocated them to either S-CST or C-CST. We examined the benefits in traditional outcomes (i.e., global cognitive functioning, mood, and psychological and behavioral symptoms), as well as in overlooked psychosocial outcomes (i.e., social and emotional loneliness) and socioemotional skills [i.e., cognitive and affective theory of mind (ToM) and definitional competence of emotions].

**Results:**

Results indicated that both C-CST and S-CST maintained PwD mood but that only S-CST supported global cognitive functioning and mitigated psychological and behavioral symptoms at postintervention. Both CST protocols also reduced social loneliness (but not emotional loneliness) and ameliorated the definitional competence of emotions at postintervention, with C-CST showing larger effect sizes compared to S-CST. Only S-CST fostered cognitive ToM (but not the affective one) at postintervention.

**Conclusion:**

Different CST protocols provided nuanced benefits across traditional, psychosocial, and socioemotional outcomes. S-CST remains the only protocol capable of promoting benefits in cognition and key dementia-related symptoms, whereas the new C-CST has emerged as a promising and easily implementable protocol with the potential to alleviate loneliness and support some socioemotional skills in PwD.

## Introduction

Cognitive Stimulation Therapy (CST) is an evidence-grounded and widely recognized psychosocial intervention for the treatment of mild-to-moderate dementia in numerous countries (Aguirre and Werheid, 2018). CST combines a cognition-based approach with psychosocial and relational features to stimulate the cognitive functioning of people with dementia (PwD) as well as to determine a broader impact on dementia-related symptoms (e.g., mood and behavioral disorders and impaired communication), in a sensitive, respectful, and person-oriented way (Kitwood, 1997). Moving beyond traditional medical models, CST adopts a holistic biopsychosocial approach that addresses not only the cognitive needs of PwD but also their overall socioemotional well-being and active involvement (Spector and Orrell, 2010). Currently recommended by the latest NICE guidelines (National Institute for Health and Care Excellence, 2018), its efficacy in supporting global cognition, language, and mood as well as in counteracting psychological and behavioral symptoms has been largely demonstrated (Desai et al., 2024; Lobbia et al., 2019; Paggetti et al., 2025; Saragih et al., 2022; Woods et al., 2023).

It is noteworthy that CST mainly involves participants in stimulating activities within small groups; however, it does not systematically rely on active collaboration among participants to complete the activities proposed. There is now increasing interest in understanding whether and how PwD can benefit from collaboration and engagement in joint activities, defined by an overarching goal or purpose that individuals share and pursue in their interaction (Clark, 1996). Preliminary evidence suggests that PwD, despite cognitive and communicative impairments, can manage a diverse range of everyday activities—even unfamiliar ones (e.g., baking (Majlesi and Ekström, 2016); using electronic devices (Ingebrand et al., 2020; Ingebrand et al., 2023))— not only in collaboration with caregivers but also with peers with dementia (Ingebrand et al., 2022). However, benefits have been generally observed with qualitative communication, engagement, and daily functioning outcomes (Majlesi and Ekström, 2016; Ingebrand et al., 2020; Ingebrand et al., 2023; Ingebrand et al., 2022).

It is also worth mentioning that, although CST and other cognitive stimulation interventions have placed emphasis on the therapeutic advantages inherent to social interactions fostered by their typical group format (Olazarán and Muñiz, 2018), to date there has been scarce evidence on whether and how such advantages could extend also to psychosocial aspects such as loneliness and socioemotional skills. Qualitative studies (Orfanos et al., 2021) have suggested that CST could alleviate loneliness [i.e., the subjective feeling of lacking relationships as a result of a perceived mismatch between desired and actual social connections (de Jong Gierveld, 1998)] that is commonly experienced among PwD (Ahadi and Hassani, 2021; Carbone et al., 2022). However, only very few studies (Atay and Bahadır Yılmaz, 2025; Capotosto et al., 2017; Piras et al., 2017) have quantitatively examined the efficacy of CST on loneliness, with inconsistent results, suggesting that it may reduce emotional loneliness (the feeling of missing close relationships) but not social loneliness (the feeling of lacking a broader network) (Weiss, 1975). There is instead a lack of evidence examining the effects of CST on PwD’s socioemotional skills related to theory of mind (ToM) and definitional competence of emotions, known to be impaired in aging and dementia (De Lucena et al., 2020; Yi et al., 2020; Bianco et al., 2022) and playing a fundamental role in supporting social functioning (Bianco et al., 2022; Eramudugolla et al., 2022; Kessels et al., 2021). ToM refers to the ability to attribute mental states to oneself and others and is essential for understanding and responding to social cues (Premack and Woodruff, 1978), encompassing both cognitive components (understanding intentions and beliefs) and affective components (understanding emotions and feelings) (Shamay-Tsoory and Aharon-Peretz, 2007). Definitional competence of emotions represents a metalinguistic ability to understand, be aware of and verbally articulate the meaning of emotions (Bianco et al., 2022; Belacchi and Benelli, 2021).

Thus, there is a lack of evidence on whether cognitively stimulating activities that require an active collaboration among PwD could be an effective approach to support their cognitive, mood, and behavioral functioning. In addition, although loneliness and socioemotional skills are closely linked to PwD’s well-being and quality of life (Ahadi and Hassani, 2021; Carbone et al., 2022; Bodden et al., 2010; Harley et al., 2008), studies assessing the efficacy of CST on such crucial aspects are still lacking. Therefore, addressing these research gaps will contribute to the understanding of CST benefits and potentially expand the application of CST in the various dementia care settings.

The first aim of the present single-blind randomized controlled trial pilot study was to evaluate the efficacy of an alternative version of CST, called Collaborative-CST (C-CST), for people with mild-to-moderate dementia, vis-à-vis the Standard CST (S-CST). S-CST involves cognitively stimulating activities that more likely require collaboration and teamwork (i.e., game-based activities entailing PwD to work together and support each other toward a common goal), which we selected from the original protocol and adapted into the new C-CST, maintaining the same duration (14 group-based sessions), structure, and person-centered approach characteristic of the S-CST. We assessed benefits in traditionally examined domains of general cognitive functioning, mood, and psychological and behavioral symptoms (Desai et al., 2024; Lobbia et al., 2019; Paggetti et al., 2025; Saragih et al., 2022; Woods et al., 2023).

Another aim was to examine the extent to which both CST protocols (C-CST and S-CST) could alleviate loneliness and support ToM and definitional competence of emotions so that we could gain new insight on the benefits of psychosocial interventions for dementia.

As for traditional outcomes, we expected that S-CST would yield greater benefits than C-CST in terms of global cognitive functioning, as it comprises a variety of activities specifically engaging multiple cognitive functions, thereby providing more comprehensive stimulation (Spector, 2018). In addition, we hypothesized both C-CST and S-CST to be effective in reducing or at least stabilizing mood and psychological and behavioral symptoms (Desai et al., 2024; Abdelkhalek et al., 2025; Coşkun and İnel Manav, 2025; Carbone et al., 2021) due to the supportive, non-judgmental environment that would allow participants to share experiences and build meaningful relationships.

Based on previous evidence (Capotosto et al., 2017), we expected both interventions to ameliorate emotional loneliness, due to activities requiring participants to engage in reciprocal interactions within a satisfying, supportive setting. For C-CST, we also expected a reduction in social loneliness, given its greater focus on teamwork and a sense of accomplishing things together.

As for ToM and definitional competence of emotions, because collaborating toward common goals may encourage participants to develop a mutual understanding of others’ perspectives and emotions, along with an enhanced ability to express and verbalize emotions (Ingebrand et al., 2022), we might expect C-SCT to be more beneficial in supporting both socioemotional skills. We could also expect similar positive effects in both CST protocols, as recalling and sharing one’s emotions, along with listening to others’ experiences in S-CST, could help PwD better understand others’ emotions and verbally articulate their own. Finally, we may also expect similar changes in definitional competence of emotions in both protocols, due to the consistent benefits in language skills observed in CST (Desai et al., 2024; Lobbia et al., 2019; Paggetti et al., 2025; Woods et al., 2023).

## Methods

This study was designed and reported following the CONSORT guidelines (Schulz et al., 2010) to ensure clarity and transparency in the methodology and presentation of results. The research was approved by the Ethical Committee of the University of Urbino Carlo Bo (N° 39-6_september_2021).

### Study design

This is a single-blind controlled clinical trial for people with mild-to-moderate dementia. A cluster randomization (Supplementary material) was performed to allocate participants in the two groups (C-CST vs. S-CST). Participants were not aware of their group allocation. A trained psychologist delivered both interventions.

### Participants

A convenience sample of PwD was recruited from six Italian residential facilities located in the province of Pesaro-Urbino following the typical inclusion criteria for CST (see Spector et al., 2006): (a) age ≥65 years; (b) a diagnosis of neurocognitive disorder according to the DSM-5 (American Psychiatric Association, 2013) in the mild-to-moderate stage, i.e., a score of ≤17 on the Montreal Cognitive Assessment (Bosco et al., 2017); (c) no history of neurodevelopmental disorders, premorbid intellectual disabilities, comorbid psychiatric disorders or physical illness/disability as well as a satisfactory ability to understand and communicate and no severe behavioral symptoms that might interfere with their participation.

Twenty-eight eligible participants were assigned either to the C-CST (*N* = 14) or to S-CST group (*N* = 14) (Supplementary material for details).

### Traditional outcomes

#### Global cognitive functioning

The *Addenbrooke’s Cognitive Evaluation-Revised* (ACE-R; (Mioshi et al., 2006; Pigliautile et al., 2012)) covers five cognitive areas: attention and orientation, memory, language, fluency, and visuospatial abilities (Cronbach’s *α* = 0.85). The dependent variable was the sum of all items, with higher scores indicating better global cognitive functioning.

#### Mood and behavior

The *Neuropsychiatric Inventory* (NPI; Binetti et al., 1998; Cummings et al., 1994) assesses the frequency and severity of 12 psychological and behavioral symptoms, including delusions, hallucinations, agitation/aggression, depression/dysphoria, anxiety, euphoria, apathy, disinhibition, irritability, aberrant motor behavior, sleep disturbances, eating disorders (Cronbach’s *α* = 0.76). The dependent variable was the sum of the frequency*severity scores from all symptoms, with higher scores corresponding to greater severity and frequency of neuropsychiatric symptoms. The *Cornell Depression Scale in Dementia* (CSDD; Alexopoulos et al., 1988) assesses depressive symptoms in PwD. It consists of 19 questions with responses on a 3-point Likert scale (Cronbach’s *α* = 0.84). The dependent variable was the sum of the scores from all items, with higher scores indicating greater severity of depressive symptoms.

### Psychosocial and socioemotional outcomes

#### Psychosocial outcome: loneliness

The *de Jong Gierveld Loneliness Scale* (dJGLS; de Jong Gierveld and Van Tilburg, 2010) is a self-report questionnaire on perceived loneliness consisting of 6 questions with responses on a 5-point Likert scale. The dependent variables were the sum of the scores for social (Cronbach’s *α* = 0.85) and emotional loneliness (Cronbach’s *α* = 0.81), with higher scores corresponding to lower perceived social and emotional loneliness, respectively.

#### Socioemotional outcomes: theory of mind

The *Reading the Mind in the Eyes Test* (RMET; Baron-Cohen et al., 2001; Preti et al., 2017) measures the ability to infer emotions based on non-verbal cues, in this case, the gaze. Participants are presented with 18 photographs of gazes taken from different actors and asked to choose the word that best describes what the person is feeling among 4 different options (Cronbach’s *α* = 0.75). The dependent variable was the sum of all correct responses, with higher scores corresponding to better affective ToM. The *Picture Sequencing Task–Short version* (PST; Bechi et al., 2012; Brüne et al., 2011) assesses cognitive ToM measuring the ability to identify the intentions and thoughts necessary to understand a social interaction and the ability to take the perspective of another person by making assumptions about his/her intentions and motivations. The test requires arranging drawings that tell a story in the correct order and answering true/false questions related to these stories (Cronbach’s *α* = 0.86). The dependent variable was the sum of all correctly ordered stories and correct true/false responses, with higher scores indicating better cognitive ToM.

#### Socioemotional outcomes: definitional competence of emotions

The *Definitional Competence Scale* (Co. De. Scale; Belacchi and Benelli, 2021) measures the ability to provide definitions of 8 emotions (fear, joy, sadness, anger, pride, envy, guilt, and shame). Answers were evaluated according to seven definitional levels, where level 0 corresponds to no response and level 6 to an Aristotelian definition (see Supplementary material for details) (Cronbach’s *α* = 0.92). The dependent variable was the mean of the definitional levels given for the considered emotions, with higher scores indicating better definitional competence of emotions.

### Procedure

All participants underwent four individual sessions—two for each of the pre- and post-test assessments—to complete a comprehensive battery of tests and questionnaires assessing the treatment’s efficacy (Table 1). All participants were then involved in 14 sessions lasting approximately 45–60 min, delivered twice a week for a total of 7 weeks in small groups (3–5 people), as recommended by the protocol (Spector et al., 2006; Gardini et al., 2015). During these sessions, participants took part in either to the Italian adaptation of the S-CST (Capotosto et al., 2017; Gardini et al., 2015) or the C-CST program.

**Table 1:** Organization of the assessment sessions and themes/activities of the sessions for the Standard-CST and the Collaborative-CST.

Pre-intervention sessions	Session 1: the ACE-R, RMET, and the dJGLS (session 1); Session 2: the Co. De. Scale and PST (session 2). The NPI and the CSDD were administered to the staff members providing daily care for the participants within the nursing home.	
	Standard-CST*	Collaborative-CST
	Main session activities engaged multiple cognitive domains (e.g., thinking, memory, problem-solving, and language) via activities tailored to the group’s interests and abilities covering, throughout the sessions, the following themes:	Main session activities empathized cooperation, turn-taking, and mutual encouragement (rather than individual performance). For the (1) Physical game – bowling: participants took turns throwing a soft foam ball at skittles; each participant’s score (number of knocked skittles) was recorded on a whiteboard and added to a shared group total score; (2) Word game – guess-the-word: the group had to guess target words (i.e., nouns) of increasing lengths shown on a whiteboard as blank spaces; one participant at a time had to provide a letter; (3) Number game – collaborative domino: participants formed a team whose shared goal was to place, one participant at a time, all domino tiles on the table, so that identical numbers touched at the chain’s open ends; (4) Team quizzes: in each session, the group had to discuss and agree on the correct answer to multiple-choice questions on various topics (history, geography, general knowledge, and popular culture) displayed on a whiteboard (e.g., “Which of these is the longest river?”). These collaborative activities were implemented throughout the sessions as follow:
Sessions (number)	Main session activities	Main session activities
1	Physical games	Physical games
2	Sounds	Word games
3	My childhood	Number games
4	Food	Quiz
5	News	Physical games
6	Faces and places	Word games
7	Word association	Number games
8	Creativity	Quiz
9	Object categorization	Physical games
10	Orientation	Word games
11	Money	Number games
12	Number games	Quiz
13	Word games	Physical games
14	Quiz	Word games
Post-intervention sessions	Session 1: the ACE-R, RMET, and the dJGLS (session 1); Session 2: the Co. De. Scale and PST (session 2). The NPI and the CSDD were administered to the staff members providing daily care for the participants within the nursing home.	

ACE-R, Addenbrooke’s Cognitive Evaluation-Revised; NPI, Neuropsychiatric Inventory; CSDD, Cornell Scale for Depression in Dementia; dJGLS, de Jong Gierveld Loneliness Scale; RMET, Reading the Mind in the Eyes Test; PST, Picture Sequencing Task; Co. De. Scale, Definitional Competence Scale.

*For a comprehensive description of the main session activities of the Standard-CST, please refer to (Spector et al., 2006).

As for the S-CST, always following the original protocol (Spector et al., 2006; Gardini et al., 2015), each session followed the same structure: (1) introduction (10 min), which included a personalized welcome; discussing a name for the group and a theme song (chosen in Session 1 and then reminded and sang in every other session); discussing the day, month, year, weather, and time, as well as the name and address of the residential center, using a whiteboard; and discussing current affairs and refreshments; (2) main sessions activities (25–40 min), covering various themes across the sessions (Table 1) and that are adapted to participants’ baseline general cognitive functioning (see Carbone et al., 2021); (3) conclusion (10 min), which included thanking everyone for attending and contributing; singing the theme song; reminding everyone of the date and time of the next session and its content; and saying goodbye.

For the C-CST group, each session mirrored the same structure used in the S-CST, with a 10-min introduction (dedicated to initial greetings, the selection of a group name and song in Session 1, and reminder in the following sessions of the group name), main session activities (25–40 min), and a 10-min conclusion (dedicated to final greetings and reminder of the next appointment). The main session activities of C-CST were derived from some of the S-CST activities that specifically promote collaboration and encourage teamwork and mutual support. Particularly, the following: (1) physical game, (2) word game, (3) number game, (4) team quizzes activities were selected, implemented and repeated across the 14 sessions (for details see Table 1).

A trained psychologist administered both interventions in a quiet room of the residential care facilities, minimizing distractions and enabling participants to concentrate on the activities.

### Statistical analyses

First, baseline differences between the S-CST and C-CST groups on sociodemographic characteristics and outcomes of interest were examined. The distribution of each continuous variable was assessed with the Shapiro–Wilk normality test, and homogeneity of variances with Levene’s test. If both assumptions were satisfied, independent-samples *t*-tests were performed; when normality held but variances were unequal, Welch’s *t*-test was applied. When normality was violated, group comparisons were conducted using the non-parametric Mann–Whitney *U* test.

Then, to assess the effects of the two CST protocols across assessment sessions, linear mixed effect models (LMEs) were conducted for each outcome of interest, with Group (S-CST vs. C-CST), Session (pre-intervention vs. post-intervention) and their interaction as predictors, and Participant (ID) as random factor. When distributional assumptions were violated, a generalized mixed model with gamma distribution was employed. To interpret the Group*Session interactions, Tukey corrected post-hoc tests were conducted.

To clarify the dimension of benefits for both the C-CST and S-CST groups, Cohen’s *d* expressing post-intervention – pre-intervention changes were calculated for each outcome. Values were corrected using the Hedges and Olkin (1985) correction factor to avoid the small sample bias.

## Results

Table 2 shows the descriptive statistics of sociodemographics and outcomes of interest by group and assessment session. No significant differences between the two groups in sociodemographics and all the outcome measures of interest at baseline emerged. Results of the LMEs are shown in Table 3. Table 4 provides effect sizes by group.

**Table 2:** Descriptive statistics of socio-demographic characteristics and measures of interest by group (Standard-CST and Collaborative-CST) and assessment session (pre-intervention and post-intervention) and results of groups comparisons at baseline.

	Baseline differences	Standard-CST (*N* = 14; 12 F)			Collaborative-CST (*N* = 14; 12 F)		
		*M*	SD	Min–Max	*M*	SD	Min–Max
Age	*t*(26) = −0.03; *p* = 0.97	87.07	6.43	77–96	87.00	5.22	78–97
Education	U = 90; *p* = 0.70	5.93	2.34	4–13	6.86	4.54	3–18
Years in residence	U = 94.5; *p* = 0.88	2.57	2.10	1–8	2.50	2.41	1–8

	Baseline differences	Pre-intervention		Post-intervention		Pre-intervention		Post-intervention	
		*M*	SD	*M*	SD	*M*	SD	*M*	SD
Traditional outcomes									
ACE-R	*t*(24) = −0.82; *p* = 0.42	60.50	7.20	68.50	7.70	58.00	8.43	58.83	8.62
NPI	*U* = 87; *p* = 0.63	18.93	9.26	18.21	9.06	20.64	11.28	22.93	12.15
CSDD	*t*(26) = −0.54; *p* = 0.59	10.43	4.29	10.86	5.10	9.57	4.03	10.64	4.05
Psychosocial and socioemotional outcomes									
dJGLS: Emotional loneliness	*t*(26) = 0.17; *p* = 0.87	10.14	1.96	10.00	3.09	10.29	2.52	10.36	2.65
dJGLS: Social loneliness	*t*(26) = −0.49; *p* = 0.63	8.79	1.97	9.21	2.08	8.43	1.91	9.79	2.22
RMET	*t*(26) = −0.07; *p* = 0.95	7.14	2.32	7.71	2.73	7.07	3.08	6.57	3.16
PST	U = 95; *p* = 0.91	2.00	1.92	3.79	2.55	2.29	2.33	2.29	2.23
Co. De. Scale	*t*(26) = −0.21; *p* = 0.84	3.13	0.49	3.46	0.33	3.08	0.65	3.82	0.79

ACE-R, Addenbrooke’s Cognitive Evaluation-Revised; NPI, Neuropsychiatric Inventory; CSDD, Cornell Scale for Depression in Dementia; dJGLS, de Jong Gierveld Loneliness Scale; RMET, Reading the Mind in the Eyes Test; PST, Picture Sequencing Task; Co. De. Scale, Definitional Competence Scale; *U*, Mann–Whitney *U* test; *t*, independent sample *t*-test.

**Table 3:** Results from mixed-effect models for the measures of interest with group (Standard-CST vs. Collaborative-CST), assessment session (pre-intervention vs. post-intervention) and their interactions as predictors.

Outcome	Predictor	Omnibus test	*B*	SE	Statistic	*p*
Traditional outcomes						
ACE-R	Group	*F*(1,24) = 4.24	6.08	2.95	*t*(24) = 2.06	0.050
	**Session**	***F*(1,24) = 17.65**	**4.42**	**1.05**	***t*(24) = 4.20**	**<0.001**
	Group*Session	***F*(1,24) = 11.61**	**7.17**	**2.10**	***t*(24) = 3.41**	**0.002**
NPI	Group	*χ*^2^(1) < 1	−3.75	4.32	*z* = −0.87	0.384
	Session	*χ*^2^(1) < 1	0.00	0.54	*z* = 0.01	0.994
	Group*Session	***χ*** ^**2**^ **(1) = 13.02**	**−3.93**	**1.09**	***z* = −3.61**	**<0.001**
CSDD	Group	*F*(1,26) < 1	0.54	1.56	*t*(26) = 0.34	0.735
	Session	*F*(1,26) = 1.83	0.75	0.56	*t*(26) = 1.35	0.188
	Group*Session	*F*(1,26) < 1	−0.64	1.11	*t*(26) = −0.58	0.568
Psychosocial and socioemotional outcomes						
dJGLS: Emotional Loneliness	Group	*F*(1,26) < 1	−0.25	0.84	*t*(26) = −0.30	0.769
	Session	*F*(1,26) < 1	−0.04	0.50	*t*(26) = −0.07	0.943
	Group*Session	*F*(1,26) < 1	−0.21	1.00	*t*(26) = −0.22	0.831
dJGLS: Social Loneliness	Group	*F*(1,26) < 1	−0.11	0.68	*t*(26) = −0.16	0.876
	**Session**	***F*(1,26) = 5.77**	**0.89**	**0.37**	***t*(26) = 2.40**	**0.024**
	Group*Session	*F*(1,26) = 1.56	−0.93	0.74	*t*(26) = −1.25	0.223
RMET	Group	*F*(1,26) < 1	0.61	0.94	*t*(26) = 0.65	0.523
	Session	*F*(1,26) < 1	0.04	0.52	*t*(26) = 0.07	0.946
	Group*Session	*F*(1,26) = 1.06	1.07	1.04	*t*(26) = 1.03	0.312
PST	Group	*χ*^2^(1) < 1	0.50	0.74	*z* = 0.67	0.505
	**Session**	***χ*** ^**2**^ **(1) = 5.96**	**0.76**	**0.31**	***z* = 2.44**	**0.015**
	Group*Session	***χ*** ^**2**^ **(1) = 8.83**	**1.84**	**0.62**	***z* = 2.97**	**0.003**
Co. De. Scale	Group	*F*(1,26) < 1	−0.16	0.18	*t*(26) = −0.85	0.404
	**Session**	***F*(1,26) = 18.38**	**0.54**	**0.13**	***t*(26) = 4.29**	**<0.001**
	Group*Session	*F*(1,26) = 2.54	−0.40	0.25	*t*(26) = −1.59	0.123

ACE-R, Addenbrooke’s Cognitive Evaluation-Revised; NPI, Neuropsychiatric Inventory; CSDD, Cornell Scale for Depression in Dementia; dJGLS, de Jong Gierveld Loneliness Scale; RMET, Reading the Mind in the Eyes Test; PST, Picture Sequencing Task; Co. De. Scale, Definitional Competence Scale. F, fixed effect omnibus test for linear mixed models; t, fixed effect parameter estimate test for linear mixed models; χ^2^, fixed effect omnibus test for generalized mixed models with gamma distribution; z, fixed effect parameter estimate test for generalized mixed models with gamma distribution. Significant results in bold.

**Table 4:** Effect sizes for pre–post benefits in standard-CST and collaborative-CST groups.

	Standard-CST	Collaborative-CST
Traditional outcomes		
ACE-R	1.03	0.09
NPI	0.08	−0.19
CSDD	−0.09	−0.25
Psychosocial and socioemotional outcomes		
dJGLS: Emotional Loneliness	−0.05	0.03
dJGLS Scale: Social Loneliness	0.20	0.63
RMET	0.21	−0.15
PST	0.76	0.00
Co. De. Scale	0.75	0.98

ACE-R, Addenbrooke’s Cognitive Evaluation-Revised; NPI, Neuropsychiatric Inventory; CSDD, Cornell Scale for Depression in Dementia; dJGLS, de Jong Gierveld Loneliness Scale; RMET, Reading the Mind in the Eyes Test; PST, Picture Sequencing Task; Co. De. Scale, Definitional Competence Scale. A positive effect size indicates an improvement in the outcome, while a negative one indicates a worsening in the outcome.

### Benefits in traditional outcomes

#### Global cognitive functioning

A significant main effect of Session emerged, with both groups showing on average a higher cognitive functioning at postintervention. The main effect of Group was not significant. A significant Group*Session interaction emerged, indicating that global cognitive functioning increased in the S-CST group and remained stable in the C-CST group at postintervention (Table 3).

#### Neuropsychiatric symptoms

A significant Group*Session interaction emerged, indicating stability over time in the S-CST group and a worsening in the C-CST group at postintervention of NPI scores. Neither the Group nor the Session main effects were significant (Table 3).

#### Mood

No significant main effects nor interaction were observed for depressive symptoms (Table 3).

### Benefits in psychosocial and socioemotional outcomes

#### Loneliness

A significant main effect of Session was found in Social Loneliness, with both groups showing reduced social loneliness at postintervention. Neither the effect of Group nor the Group*Session interaction was significant.

No significant main effects nor interactions were observed for Emotional Loneliness (Table 3).

#### Theory of mind

As for affective ToM, no significant main effects nor interactions were observed for affective ToM (Table 3).

Regarding cognitive ToM, a significant main effect of Session emerged: on average, both groups showed an increased ability to interpret other people’s intention at postintervention. A significant Group*Session interaction emerged, indicating that the ability to interpret other people’s intention increased significantly in the S-CST group, but did not significantly vary in the C-CST group at postintervention. No other main effects emerged (Table 3).

#### Definitional competence of emotions

A significant main effect of Session emerged for the Co. De. Scale, with both groups showing more refined definitional competence of emotions at postintervention. Neither the effect of Group nor the Group*Session interaction was significant (Table 3).

#### Evaluation of interventions impact via effect sizes

Concerning effect sizes (Table 4), for ACE-R effect sizes were large for S-CST and negligible for C-CST. As for NPI and CSDD, effect sizes were small-to-negligible for both groups. Effect sizes were small for S-CST and medium for C-CST for Social Loneliness, whereas negligible for both groups for Emotional Loneliness. For cognitive ToM (PST), effect sizes were medium for S-CST and negligible for C-CST, whereas small-to-negligible effect size emerged for affective ToM (RMET). For Co. De., effect sizes were medium for S-CST and large for C-CST.

## Discussion

This pilot study evaluated the benefits of a new CST version, focused more on collaborative activities (C-CST), as compared to the standard protocol (S-CST), on global cognitive functioning, mood, and psychological and behavioral symptoms. We also explored the extent to which S-CST and C-CST also benefit loneliness—a psychosocial outcome rarely explored in PwD—and, for the first time in the CST context, socioemotional skills (i.e., ToM and a metalinguistic ability as the definitional competence of emotions).

Regarding traditional outcomes, S-CST was confirmed to be effective in sustaining global cognitive functioning, consistent with the latest systematic review (Desai et al., 2024; Lobbia et al., 2019; Paggetti et al., 2025; Saragih et al., 2022; Woods et al., 2023). Moreover, as hypothesized, S-CST provided greater benefits in terms of global cognitive functioning than C-CST. Such an advantage could lie in the fact that S-CST entails a broad range of activities, arranged to engage multiple cognitive domains (Clare and Woods, 2004) and proposed following the “choice” and “maximizing potential” key principles, enhancing the protocol efficacy in terms of supporting global cognition (Olazarán and Muñiz, 2018). C-CST instead focused on teamwork and joint problem-solving, but with fewer varied activities; this might have prevented the broader cognitive stimulation and the opportunity to engage in activities that match abilities and potential, which represent crucial aspects for supporting global cognitive functioning in PwD (Olazarán and Muñiz, 2018; de Werd et al., 2013).

Our results showed no changes in depressive symptoms after both S-CST and C-CST; this is partially in line with our expectations, but consistent with previous evidence on CST (Abdelkhalek et al., 2025; Carbone et al., 2021) supporting that no changes (i.e., maintenance) more than not a decrease over time in mood—can be viewed as a positive outcome in dementia care (Lobbia et al., 2019). These findings highlight how an “enriched environment” in terms of cognitively and socially stimulating experiences, as well as interactions prompted by both CST programs, can provide emotional support (Yun et al., 2020), along with a preserved sense of identity (Goodall et al., 2018).

As for NPI, S-CST, but not C-CST, helped stabilize psychological and behavioral symptoms. Such a pattern of findings might be due to the key principles and varied activities characteristic of S-CST, which were not comprehensively integrated into C-CST. S-CST offered structured, enjoyable activities in a supportive environment, encouraging personal expression rather than factual correctness (Spector, 2018), aspects that could reduce frustration, ensure emotional safety, and, in turn, possibly mitigate challenging behaviors by fostering personhood (Coşkun and İnel Manav, 2025; Carbone et al., 2021). In contrast, C-CST emphasized task correctness and collective goals, which might lead to frustration or disengagement, limiting opportunities for opinion-based responses and thereby making it difficult to stabilize psychological and behavioral symptoms.

Regarding psychosocial outcomes, both CST protocols reduced social loneliness, partially in line with our hypothesis. Both interventions promote psychosocial interaction and offer a sense of routine that can help participants feel more socially engaged and supported by their peers, mitigating their sense of meaninglessness (Telenius et al., 2022). Interestingly, the effect size for social loneliness was small in S-CST, whereas it was medium in C-CST, indicating a trend favoring a focus on collaboration as being more effective in addressing the feeling of missing a wider social network. Possibly, engaging PwD in joint activities is likely to foster a greater sense of connection and shared experience, enhancing their perceived social support and reducing feelings of isolation. However, no changes were observed in emotional loneliness after both CST protocols. Such findings are contrary to previous evidence (Capotosto et al., 2017) and our expectations, which might be due to differences in the residential facility’s characteristics (e.g., care approach and facility size) or opportunities for contact with family members that could affect emotional support and interact with CST effects, which are aspects that deserve further investigation.

As for socioemotional skills, engaging in both CST protocols did not benefit the understanding of others’ emotions (affective ToM), contrary to our expectations. Such a result could lie in the fact that neither protocol specifically targets emotional understanding, focusing instead on general cognitive and social stimulation. These protocols may not have been adapted to produce changes in this domain, as the development of ToM skills often requires more specific activities in aging (Cavallini et al., 2015). Nonetheless, S-CST, but not C-CST, improved cognitive ToM. It could be that the benefits obtained by this group in cognitive functioning may also have provided the basis for an effect in the cognitive dimension of ToM, supporting the understanding of others’ intentions (Moran, 2013). Another explanation could be that understanding others requires self-reflection, particularly on autobiographical memories and past experiences—features that characterize S-CST activities but were absent in C-CST. Similarly, understanding others often requires familiarity with them, which may occur more naturally in S-CST, where participants engage more deeply with each other’s personal stories during group discussions, fostering a better comprehension of others’ viewpoints. These speculations, which deserve further investigation, align with evidence suggesting that PwD rely on their own past experiences to infer the mental states of others (Moreau et al., 2013).

Interestingly, both CST protocols benefited the definitional competence of emotions, with PwD showing higher definitional levels for the proposed emotions after the intervention. CST seems to improve the knowledge of one’s emotions in relation to others and the ability to communicate this knowledge, thanks to the experience of being in a group and the consequent need to use language to understand others and make oneself understood. Among the various domains that CST targets, language appears to benefit particularly, as highlighted in numerous studies (Desai et al., 2024; Lobbia et al., 2019; Paggetti et al., 2025); therefore, the enhanced competence of verbally defining emotions could be partially due to this effect. The effect size was medium for S-CST and large for C-CST, indicating a trend suggesting that a greater focus on collaboration may enhance the definitional competence of emotions. Thus, it could be speculated that working toward a common goal helps PwD share their emotions and become more attuned to the emotions of others, facilitating a higher level of knowledge of them.

Despite these promising findings, some limitations should be acknowledged. First, as a pilot study, it includes a small sample size, limiting both the robustness and generalizability of the findings. Moreover, the absence of a follow-up assessment prevented us from determining whether the benefits observed from both CST protocols were sustained over time. It is also worth mentioning that, although S-CST’s efficacy is well established, further research is needed to optimize the duration, frequency, and contents of C-CST. For instance, future studies should explore additional collaborative activities targeting more cognitive domains while maintaining CST’s person-centered approach to better investigate benefits in psychosocial and socioemotional outcomes. Another relevant unresolved issue concerning both S-CST and C-CST is the possibility of determining whether any benefits provided stand to the cognitively stimulating activities *per se* and/or to the psychosocial and relational features inherent to the group setting in which the intervention is delivered (Clare and Woods, 2004). Some studies indeed suggest that group setting plays a significant role in the benefits of CST (Orfanos et al., 2021), but the exploratory nature of our pilot study did not allow us to disentangle the contribution of these two components. Further research, also considering a more comprehensive battery of psychosocial and socioemotional measures, is therefore needed to clarify the underlying mechanisms of CST benefits, as well as to better understand the extent to which factors related to social interaction contribute to the benefits of CST.

Nonetheless, our findings carry practical implication toward selecting and implementing, in various contexts (e.g., residential care settings, day-care settings, and territorial care), different psychosocial intervention approaches based on cognitive stimulation depending on the needs and characteristics of PwD as well as the care purposes. S-CST emerges as the more convenient intervention approach for counteracting cognitive decline, stabilizing psychological and behavioral symptoms, and, as reported here for the first time, supporting the understanding of others’ intentions and opinions. Therefore, to maximize cognitive benefits and counteract mood and behavioral symptoms, interventions should prioritize diversified activities that stimulate residual cognitive abilities, encourage personal expression, and emphasize individual opinions and strengths rather than just task performance. Differently, C-CST is an easily implementable protocol that can be particularly valuable in contexts where social interaction is a key therapeutic goal and may benefit participants who thrive in environments with measurable success. Collaboration encourages active participation and communication, and it strengthens social bonds, reducing feelings of isolation. C-CST is well-suited for individuals who can still engage in problem-solving and teamwork, making it a good fit for those in earlier dementia stages. The simplicity of the C-CST protocol enhances its adaptability across various care settings, providing flexibility that is particularly valuable in resource-limited environments or in situations where maintaining consistent and engaging activities for participants is a priority.

## Conclusion

This pilot study highlights how incorporating diversified activities in cognitive stimulation programs for PwD could lead to nuanced benefits in traditional outcomes as well as psychosocial and socioemotional ones. C-CST maintains mood, and more likely alleviates social loneliness and supports socioemotional aspects related to definitional competence of emotions. Nonetheless, S-CST confirms its efficacy in supporting cognitive functioning and socioemotional aspects related to ToM, while counteracting psychological and behavioral symptoms and maintaining mood, whereby remaining the best option for providing more comprehensive care for PwD.

## Data Availability

The raw data supporting the conclusions of this article will be made available by the authors, without undue reservation.

## References

[ref1] Abdelkhalek H. Elliott K. Whitfield T. Pazvantova K. Zabihi S. Wenborn J.. (2025). Effectiveness of a 14-week protocol for cognitive stimulation therapy for mild dementia: results from a pragmatic study using routinely collected clinical data. Aging Ment. Health 29, 549–557. doi: 10.1080/13607863.2024.2410256 39364856

[ref2] Aguirre E. Werheid K. (2018). “Guidelines for adapting cognitive stimulation therapy to other cultures” in Cognitive stimulation therapy for dementia: history, evolution and internationalism. eds. Yates L. Yates J. A. Orrell M. Spector A. Woods B. (New York: Routledge), 177–193.

[ref3] Ahadi B. Hassani B. (2021). Loneliness and quality of life in older adults: the mediating role of depression. Ageing Int. 46, 337–350. doi: 10.1007/s12126-021-09408-y

[ref4] Alexopoulos G. S. Abrams R. C. Young R. C. Shamoian C. (1988). The Cornell scale for depression in dementia: Administration & Scoring guidelines. Biol. Psychiatry 23, 271–284. doi: 10.1016/0006-3223(88)90038-8 3337862

[ref5] American Psychiatric Association (2013). Diagnostic and statistical manual of mental disorders. 5th Edn. Arlington, VA: American Psychiatric Publishing.

[ref6] Atay E. Bahadır Yılmaz E. (2025). The effect of cognitive stimulation therapy (CST) on apathy, loneliness, anxiety and activities of daily living in older people with Alzheimer’s disease: randomized control study. Aging Ment. Health 29, 897–905. doi: 10.1080/13607863.2024.2437060, PMID: 39668706

[ref7] Baron-Cohen S. Wheelwright S. Hill J. Raste Y. Plumb I. (2001). The “Reading the mind in the eyes” test revised version: a study with Normal adults, and adults with Asperger syndrome or high-functioning autism. J. Child Psychol. Psychiatry Allied Discip. 42, 241–251. doi: 10.1111/1469-7610.00715 11280420

[ref8] Bechi M. Riccaboni R. Ali S. Fresi F. Buonocore M. Bosia M.. (2012). Theory of mind and emotion processing training for patients with schizophrenia: preliminary findings. Psychiatry Res. 198, 371–377. doi: 10.1016/j.psychres.2012.02.004, PMID: 22425473

[ref9] Belacchi C. Benelli B. (2021). Valutare la competenza definitoria. La Scala Co.De. in ambito clinico e nello sviluppo tipico. Milan, Italy: Franco Angeli.

[ref10] Bianco F. Castelli I. Belacchi C. (2022). Changes of meta-representational skills in ageing: first empirical evidence on the relation between metalinguistic competence and attributions of mental states. J. Lang. Educ. 8, 40–51. doi: 10.17323/jle.2022.13868

[ref11] Binetti G. Mega M. S. Magni E. Padovani A. Rozzini L. Bianchetti A.. (1998). Behavioral disorders in Alzheimer disease: a transcultural perspective. Arch. Neurol. 55, 539–544. doi: 10.1001/archneur.55.4.539, PMID: 9561983

[ref12] Bodden M. E. Mollenhauer B. Trenkwalder C. Cabanel N. Eggert K. M. Unger M. M.. (2010). Affective and cognitive theory of mind in patients with Parkinson’s disease. Parkinsonism Relat. Disord. 16, 466–470. doi: 10.1016/j.parkreldis.2010.04.014, PMID: 20538499

[ref13] Bosco A. Spano G. Caffò A. O. Lopez A. Grattagliano I. Saracino G.. (2017). Italians do it worse. Montreal cognitive assessment (MoCA) optimal cut-off scores for people with probable Alzheimer’s disease and with probable cognitive impairment. Aging Clin. Exp. Res. 29, 1113–1120. doi: 10.1007/s40520-017-0727-6, PMID: 28155182

[ref14] Brüne M. Schaub D. Juckel G. Langdon R. (2011). Social skills and behavioral problems in schizophrenia: the role of mental state attribution, neurocognition and clinical symptomatology. Psychiatry Res. 190, 9–17. doi: 10.1016/j.psychres.2010.03.015, PMID: 20417974

[ref15] Capotosto E. Belacchi C. Gardini S. Faggian S. Piras F. Mantoan V.. (2017). Cognitive stimulation therapy in the Italian context: its efficacy in cognitive and non-cognitive measures in older adults with dementia. Int. J. Geriatr. Psychiatry 32, 331–340. doi: 10.1002/gps.4521, PMID: 27272538

[ref16] Carbone E. Gardini S. Pastore M. Piras F. Vincenzi M. Borella E. (2021). Cognitive stimulation therapy for older adults with mild-to-moderate dementia in Italy: effects on cognitive functioning, and on emotional and neuropsychiatric symptoms. J Gerontol Series B 76, 1700–1710. doi: 10.1093/geronb/gbab007, PMID: 33437991

[ref17] Carbone E. Piras F. Pellegrini F. F. Caffarra P. Borella E. (2022). Individual differences among older adults with mild and moderate dementia in social and emotional loneliness and their associations with cognitive and psychological functioning. BMC Geriatr. 22:859. doi: 10.1186/s12877-022-03517-2, PMID: 36380269 PMC9667624

[ref18] Cavallini E. Bianco F. Bottiroli S. Rosi A. Vecchi T. Lecce S. (2015). Training for generalization in theory of mind: a study with older adults. Front. Psychol. 6:1123. doi: 10.3389/fpsyg.2015.01123, PMID: 26300818 PMC4523701

[ref19] Clare L. Woods R. T. (2004). Cognitive training and cognitive rehabilitation for people with early-stage Alzheimer's disease: a review. Neuropsychol. Rehabil. 14, 385–401. doi: 10.1080/09602010443000074

[ref20] Clark H. H. (1996). Using language. Cambridge: Cambridge University Press.

[ref21] Coşkun E. İnel Manav A. (2025). Evaluation of the effects of cognitive stimulation therapy on cognitive status and apathy in older adults with mild cognitive impairment: A randomized controlled trial: Applied Neuropsychology: Adult, 1–12. 10.1080/23279095.2025.2527732 40637155

[ref22] Cummings J. L. Mega M. Gray K. Rosenberg-Thompson S. Carusi D. A. Gornbein J. (1994). The neuropsychiatric inventory: comprehensive assessment of psychopathology in dementia. Neurology 44:2308. doi: 10.1212/WNL.44.12.2308, PMID: 7991117

[ref23] de Jong Gierveld J. (1998). A review of loneliness: concept and definitions, determinants and consequences. Rev. Clin. Gerontol. 8, 73–80. doi: 10.1017/S0959259898008090

[ref24] de Jong Gierveld J. Van Tilburg T. (2010). The de Jong Gierveld short scales for emotional and social loneliness: tested on data from 7 countries in the UN generations and gender surveys. Eur. J. Ageing 7, 121–130. doi: 10.1007/s10433-010-0144-6, PMID: 20730083 PMC2921057

[ref25] De Lucena A. T. Bhalla R. K. Belfort Almeida Dos Santos T. T. Dourado M. C. N. (2020). The relationship between theory of mind and cognition in Alzheimer’s disease: a systematic review. J. Clin. Exp. Neuropsychol. 42, 223–239. doi: 10.1080/13803395.2019.1710112, PMID: 31902277

[ref26] de Werd M. M. Boelen D. Rikkert M. G. O. Kessels R. P. (2013). Errorless learning of everyday tasks in people with dementia. Clin. Interv. Aging 8, 1177–1190. doi: 10.2147/CIA.S46809, PMID: 24049443 PMC3775624

[ref27] Desai R. Leung W. G. Fearn C. John A. Stott J. Spector A. (2024). Effectiveness of cognitive stimulation therapy (CST) for mild to moderate dementia: a systematic literature review and meta-analysis of randomised control trials using the original CST protocol. Ageing Res. Rev. 97:102312. doi: 10.1016/j.arr.2024.102312, PMID: 38636561

[ref28] Eramudugolla R. Huynh K. Zhou S. Amos J. G. Anstey K. J. (2022). Social cognition and social functioning in MCI and dementia in an epidemiological sample. J. Int. Neuropsychol. Soc. 28, 661–672. doi: 10.1017/S1355617721000898, PMID: 34486512

[ref29] Gardini S. Pradelli S. Faggian S. Borella E. (2015). La terapia di stimolazione cognitiva: un intervento efficace per la persona con demenza. Milan, Italy: Franco Angeli.

[ref30] Goodall G. Ciobanu I. Broekx R. Sørgaard J. Anghelache I. Anghelache-Tutulan C.. (2018). Using adaptive immersive environments to stimulate emotional expression and connection in dementia care. In Fourth International Conference on Human and Social Analytics.

[ref31] Harley T. A. Jessiman L. J. MacAndrew S. B. Astell A. (2008). I don't know what I know: evidence of preserved semantic knowledge but impaired metalinguistic knowledge in adults with probable Alzheimer's disease. Aphasiology 22, 321–335. doi: 10.1080/02687030701391065

[ref32] Hedges L. V. Olkin I. (1985). Statistical methods for Meta-Analysis. Orlando: Academic Press. doi: 10.1080/17483107.2020.1800117

[ref33] Ingebrand E. Samuelsson C. Hydén L. C. (2020). A person living with dementia learning to navigate an iPad: a case study. Disabil. Rehabil. Assist. Technol. 17, 570–579. 32757964 10.1080/17483107.2020.1800117

[ref34] Ingebrand E. Samuelsson C. Hydén L. C. (2022). People living with dementia collaborating in a joint activity. Learn. Cult. Soc. Interact. 34:100629. doi: 10.1016/j.lcsi.2022.100629

[ref35] Ingebrand E. Samuelsson C. Hydén L. C. (2023). Supporting people living with dementia in novel joint activities: managing tablet computers. J. Aging Stud. 65:101116. doi: 10.1016/j.jaging.2023.101116, PMID: 37268389

[ref36] Kessels R. P. Waanders-Oude Elferink M. van Tilborg I. (2021). Social cognition and social functioning in patients with amnestic mild cognitive impairment or Alzheimer’s dementia. J. Neuropsychol. 15, 186–203. doi: 10.1111/jnp.12223, PMID: 32979297 PMC8247057

[ref37] Kitwood T. (1997). Dementia reconsidered: the person comes first. Buckingham, UK: Open University Press.

[ref38] Lobbia A. Carbone E. Faggian S. Gardini S. Piras F. Spector A.. (2019). The efficacy of cognitive stimulation therapy (CST) for people with mild-to-moderate dementia. Eur. Psychol. 24, 257–277. doi: 10.1027/1016-9040/a000342

[ref39] Majlesi A. R. Ekström A. (2016). Baking together—the coordination of actions in activities involving people with dementia. J. Aging Stud. 38, 37–46. doi: 10.1016/j.jaging.2016.04.004, PMID: 27531451

[ref40] Mioshi E. Dawson K. Mitchell J. Arnold R. Hodges J. R. (2006). The Addenbrooke's cognitive examination revised (ACE-R): a brief cognitive test battery for dementia screening. Int. J. Geriatr. Psychiatry 21, 1078–1085. doi: 10.1002/gps.1610, PMID: 16977673

[ref41] Moran J. M. (2013). Lifespan development: the effects of typical aging on theory of mind. Behav. Brain Res. 237, 32–40. doi: 10.1016/j.bbr.2012.09.020, PMID: 23000532

[ref42] Moreau N. Viallet F. Champagne-Lavau M. (2013). Using memories to understand others: the role of episodic memory in theory of mind impairment in Alzheimer disease. Ageing Res. Rev. 12, 833–839. doi: 10.1016/j.arr.2013.06.005, PMID: 23838323

[ref43] National Institute for Health and Care Excellence. (2018). Dementia: assessment, management and support for people living with dementia and their carers. Available at: https://www.nice.org.uk/guidance/ng97/chapter/Recommendations#interventions-to-promote-cognition-independence-and-wellbeing (Accessed April 7, 2025). 30011160

[ref44] Olazarán J. Muñiz R. (2018). “Cognitive stimulation, training, and rehabilitation: the bigger picture” in Cognitive stimulation therapy for dementia: history, evolution and internationalism. eds. Yates L. Yates J. A. Orrell M. Spector A. Woods B. (New York: Routledge), 11–30.

[ref45] Orfanos S. Gibbor L. Carr C. Spector A. (2021). Group-based cognitive stimulation therapy for dementia: a qualitative study on experiences of group interactions. Aging Ment. Health 25, 991–998. doi: 10.1080/13607863.2020.1746740, PMID: 32272849

[ref46] Paggetti A. Druda Y. Sciancalepore F. Della Gatta F. Ancidoni A. Locuratolo N.. (2025). The efficacy of cognitive stimulation, cognitive training, and cognitive rehabilitation for people living with dementia: a systematic review and meta-analysis. GeroScience 47, 409–444. doi: 10.1007/s11357-024-01400-z, PMID: 39485657 PMC11872969

[ref47] Pigliautile M. Ricci M. Mioshi E. Ercolani S. Mangialasche F. Monastero R.. (2012). Validation study of the Italian Addenbrooke’s cognitive examination revised in a young-old and old-old population. Dement. Geriatr. Cogn. Disord. 32, 301–307. 10.1159/000334657 22262124

[ref48] Piras F. Carbone E. Faggian S. Salvalaio E. Gardini S. Borella E. (2017). Efficacy of cognitive stimulation therapy for older adults with vascular dementia. Dementia Neuropsychol 11, 434–441. doi: 10.1590/1980-57642016dn11-040014, PMID: 29354225 PMC5770003

[ref49] Premack D. Woodruff G. (1978). Does the chimpanzee have a theory of mind? Behav. Brain Sci. 1, 515–526. doi: 10.1017/S0140525X00076512

[ref50] Preti A. Vellante M. Petretto D. R. (2017). The psychometric properties of the “Reading the mind in the eyes” test: an item response theory (IRT) analysis. Cognit. Neuropsychiatry 22, 233–253. doi: 10.1080/13546805.2017.1300091 28288549

[ref51] Saragih I. D. Tonapa S. I. Saragih I. S. Lee B. O. (2022). Effects of cognitive stimulation therapy for people with dementia: a systematic review and meta-analysis of randomized controlled studies. Int. J. Nurs. Stud. 128:104181. doi: 10.1016/j.ijnurstu.2022.104181, PMID: 35149325

[ref52] Schulz K. F. Altman D. G. Moher D. CONSORT Group* (2010). CONSORT 2010 statement: updated guidelines for reporting parallel group randomized trials. Ann. Intern. Med. 152, 726–732. doi: 10.7326/0003-4819-152-11-201006010-00232, PMID: 20335313

[ref53] Shamay-Tsoory S. G. Aharon-Peretz J. (2007). Dissociable prefrontal networks for cognitive and affective theory of mind: a lesion study. Neuropsychologia 45, 3054–3067. doi: 10.1016/j.neuropsychologia.2007.05.021, PMID: 17640690

[ref54] Spector A. (2018). “Introduction” in Cognitive stimulation therapy for dementia: history, evolution and internationalism. eds. Yates L. Yates J. A. Orrell M. Spector A. Woods B. (New York: Routledge), 3–10.

[ref55] Spector A. Orrell M. (2010). Using a biopsychosocial model of dementia as a tool to guide clinical practice. Int. Psychogeriatr. 22, 957–965. doi: 10.1017/S1041610210000840, PMID: 20561384

[ref56] Spector A. Thorgrimsen L. Woods R. T. Orrell M. (2006). Making a difference: an evidence-based group programme to offer cognitive stimulation therapy (CST) to people with dementia. Wimbledon: Hawker Publications.

[ref57] Telenius E. W. Tangen G. G. Eriksen S. Rokstad A. M. M. (2022). Fun and a meaningful routine: the experience of physical activity in people with dementia. BMC Geriatr. 22:500. doi: 10.1186/s12877-022-03149-6, PMID: 35689197 PMC9188090

[ref58] Weiss R. (1975). Loneliness: the experience of emotional and social isolation. Cambridge, MA: MIT Press.

[ref59] Woods B. Rai H. K. Elliott E. Aguirre E. Orrell M. Spector A. (2023). Cognitive stimulation to improve cognitive functioning in people with dementia. Cochrane database Syst Rev 2023:CD005562. doi: 10.1002/14651858.CD005562.pub2 PMC9891430 39804128

[ref60] Yi Z. Zhao P. Zhang H. Shi Y. Shi H. Zhong J.. (2020). Theory of mind in Alzheimer’s disease and amnestic mild cognitive impairment: a meta-analysis. Neurol. Sci. 41, 1027–1039. doi: 10.1007/s10072-019-04215-5, PMID: 31912336

[ref61] Yun S. J. Kang M. G. Yang D. Choi Y. Kim H. Oh B. M.. (2020). Cognitive training using fully immersive, enriched environment virtual reality for patients with mild cognitive impairment and mild dementia: feasibility and usability study. JMIR Serious Games 8:e18127. doi: 10.2196/18127, PMID: 33052115 PMC7593866

