# Supplementary material for: Comparing the benefits of Cognitive Stimulation Therapy with an adapted collaborative version on traditional outcomes, loneliness, and socioemotional skills: a pilot study
Source: Front Psychol. 2025 Sep 10;16:1607552. doi: 10.3389/fpsyg.2025.1607552 (PMC12457668; doi:10.3389/fpsyg.2025.1607552)
Supplement: Supplementary file 1 [file Data_Sheet_1.docx]

**SUPPLEMENTARY MATERIAL**

**Comparing the benefits of Cognitive Stimulation Therapy with an adapted collaborative version on traditional outcomes, loneliness, and socioemotional skills: a pilot study**

**Randomization process details**

Participants were assigned to the two groups (Standard-CST vs. Collaborative-CST) through cluster randomization to ensure groups of at least three participants that took part in the two interventions within each residential facility. This approach was chosen due to the small number of participants per facility, avoiding issues of insufficient group sizes that would arise with individual-level randomization. Cluster randomization ensured that all participants within a cluster received the same treatment, facilitating consistent group-based interventions (Donner, 1998). Clusters of 3 or 4 participants were created within individual facilities as homogeneous as possible for the following variables: age, education, and MoCA score in the preintervention phase. These clusters were then assigned to the two intervention groups using the R package *randomizr* (Uschner et al., 2018). 5 clusters (N = 18) were assigned to the Standard-CST group, while 5 clusters (N = 18) were assigned to the Collaborative-CST group.

Initially, 36 eligible participants were recruited.

All participants underwent the initial assessment, however 8 participants dropped out before the post-intervention phase due to death (1 participant), moving to another facility (2), unwillingness to continue participating (5).

**Co.De. Scale details** (adapted from Belacchi & Benelli, 2021)

Eight emotion-related nouns, were presented: fear, joy, sadness, anger, pride, envy, guilt, and shame. The scale is divided into seven defining levels (from 0 to 6), where level 0 corresponds to a lack of response and level 6 corresponds to an Aristotelian definition, i.e., a categorizing periphrastic definition with discriminating specification (see Supplementary Table 2 for an illustration of the response levels). To reach the highest level of metalinguistic definition, it is necessary for the statement to respect both the constitutive rules of definitions and to present an Aristotelian structure ("x is a y that z").

Participants are asked to provide the meaning of the word (prompt: "*What does the word X mean?*"); in case of difficulty understanding the question, a more pragmatically oriented request is made, such as "*imagine explaining this word to someone who does not know its meaning*." The participant responds orally, and each response is transcribed verbatim; among the possible different responses to a stimulus word, the score of the highest-level response is considered.

**Supplementary Table 2.** Levels and defining rules of the Co.De. Scale.

| **SCALE LEVELS AND DEFINING PROPERTIES** | **RULES CHARACTERIZING DIFFERENT LEVELS** |
| --- | --- |
| LEVEL 0  NON-DEFINITION – score 0  No answer or non-verbal answers | Formal rule: Absence of verbal language for identifying the meaning (exclusive use of descriptive or representative gestures).  Content rule: Either complete absence of representation (no response or "I don't know" responses) or content presented implicitly in non-verbal form. |
| LEVEL I  PRE-DEFINITION – score 1  Verbal answer (one-word answers)  Es. joy 🡪 “*happy*” | Formal rule: Defining a word requires the use of words (even mere repetition of the stimulus word = tautology) and not gestures.  Content rule: The content can also be incorrect. |
| LEVEL II  NEARLY-DEFINITION – score 2  Initial formulation of sentences, without autonomous forms  Es. joy 🡪 “*when you are happy*” | Formal rule: Defining involves extending the verbal answer to expand the content. Tautology may be present. Formal inadequacy does not allow for effective elucidation of meaning.  Content rule: The content is usually subjective-experiential in nature and can be correct or incorrect. |
| LEVEL III  NARRATIVE/DESCRIPTIVE DEFINITION – score 3  Formally correct and autonomous sentences  Es. joy 🡪 “*it’s good*” | Formal Rule: Structural autonomy and morphosyntactic correctness are the formal and expressive requirements that allow accessing the meaning of the word, which would otherwise remain ambiguous. Tautologies may be present.  Content Rule: The content, which can also be incorrect, still focuses on concrete/experiential aspects. |
| LEVEL IV  SIMPLE CATEGORICAL DEFINITION – score 4  Formally correct and autonomous sentences in simple categorical/synonymic form  Es. joy 🡪 “*happiness*” | Formal Rule: Use of one-word-answers, but consisting of terms of a Superordinate or Synonymous type syntactically linked to the word-to-define by the copula ("An X is a Y" = minimum structure of the Aristotelian formula).  Content Rule: The content can still be incorrect, but it introduces a level of general-abstract consideration that identifies the conventional taxonomy or conceptual-semantic scope of reference. |
| LEVEL V  PARTIAL ARISTOTELIAN DEFINITION – score 5  Formal correctness without semantic equivalence  Es. joy 🡪 “*it’s a good thing*” | Formal Rule: The complete Aristotelian formula is present: "X is a Y that Z". The Superordinate or Synonymous term must be in the first position in the answer, complying with the principle of maximum informational relevance, to delimit the scope of possible predications about the word. The specifications are not yet fully discriminating. Tautologies may still be present.  Content Rule: The expressed content does not allow the unequivocal identification of the meaning of the word, as it can be either entirely incorrect or incomplete. |
| LEVEL VI  ARISTOTELIAN DEFINITION – score 6  Formal and semantic correctness and equivalence  Es. joy 🡪 “*it’s a positive emotion of happiness*” | Formal Rule: Use of the Aristotelian formula: "X is a Y that Z".  Content Rule: The content is always correct and allows for the unequivocal identification of the meaning, through the adequate informativeness of the specifications added to the appropriate introductory terms of different degrees of abstraction. At this level, tautology cannot be present anymore. It achieves the integration between formal correctness and content correctness. |

**References**

Belacchi, C., & Benelli, B. (2021). *Valutare la competenza definitoria. La Scala Co.De. in ambito clinico e nello sviluppo tipico*. Franco Angeli, Milan, Italy.

Donner, A. (1998). Some aspects of the design and analysis of cluster randomization trials. *Journal of the Royal Statistical Society: Series C (Applied Statistics)*, 47(1), 95-113.

Uschner, D., Schindler, D., Hilgers, R., & Heussen, N. (2018). randomizeR: an R package for the assessment and implementation of randomization in clinical trials. *Journal of Statistical Software*, 85, 1–22.
